# The complete chloroplast genome of Endangered species *Aristolochia delavayi* Franch. (Aristolochiaceae) in Southwestern China

**DOI:** 10.1080/23802359.2021.1931506

**Published:** 2021-08-01

**Authors:** Jing Zhao, Xiang-Li Yue, Zhao-Rong He, Xin-Mao Zhou

**Affiliations:** a School of Life and Science, Yunnan University, Kunming, China; b School of Ecology and Environmental Science, Yunnan University, Kunming, China

**Keywords:** IUCN, *Aristolochia manshuriensis*, phylogeny, Endangered plant

## Abstract

The complete chloroplast genome of *Aristolochia delavayi* was determined in this study. The chloroplast genome consists of 160,344 bp, with a typical circular structure including a pair of inverted repeats of 25,454 bp separated by a large single-copy region and a small single-copy region of 89,502 and 19,795 bp, respectively. The plastome contains 130 genes, including 85 protein-coding genes, eight rRNA genes, and 37 tRNA genes. Further phylogenetic analyses were conducted using 12 complete plastomes of *Aristolochia*. These data support a close relationship between *Aristolochia delavayi* and *Aristolochia tubiflora*.

*Aristolochia delavayi* Franch (1898: 12) is a perennial herbal and endemic to the dry and hot valley of Jingshajiang River in the northwest Yunnan and the southwest Sichuan provinces of China (Li et al. 2013). *A. delavayi* is the member of family Aristolochiaceae. Its leaves and fruits were used for spices and often cooked with beef and mutton by the local people in Yunnan (Yang et al. 2014). Artificial collections, construction of hydropower stations, and changes in the ecological environment have seriously threatened this species (Yang et al. 2014). Based on IUCN Red List Criteria (the International Union for Conservation of Nature and Natural Resources), *A. delavayi* has been classified as Endangered (EN) (China Plant Specialist Group 2004). It has been also collected in Study and Conservation of Plant Species with Extremely Small Populations (PSESP) in Yunnan Province, China (Sun et al. 2019).

Leaf tissue of *A. delavayi* was collected from Zhongdian County, Yunnan (27.310273N, 100.21103E; voucher: X.M. Zhou and J. Zhao PYU-S-911) in China, and specimens were deposited at herbarium PYU (herbarium acronyms follow Index Herbariorum by Thiers 2018).

Total DNA of *Aristolochia delavayi* was extracted from silica-dried material using the TIANGEN plant genomic DNA extraction kit (TIANGEN Biotech., Beijing, China) following the manufacturers’ protocols. An Illumina library kit was applied to an Illumina flow cell for cBOT cluster generation. Sequencing was performed on the Illumina HiSeq instrument. Illumina paired-end sequencing generated raw reads (31,047,386-bp) after removing adapters in Illumina HiSeq 2500 platform at the Beijing Novogene Technology Co., Ltd. (Tianjin, China), which had been deposited in the NCBI Sequence Read Archive (SRA: PRJNA704482).

To assemble the chloroplast of *Aristolochia delavayi*, Illumina paired-end reads were initially mapped to the chloroplast sequences of *A. manshuriensis*. The mapped reads were assembled into contigs using the GetOrganelle (Jin et al. 2020). The contigs were imported into BANDAGE v. 0.8.1 to visualize and analyze the assembly by importing the fastg file created by GetOrganelle (Wick et al. 2015). The chloroplast was annotated using Geneious R9.1.4 (Biomatters Ltd., Auckland, New Zealand) against the chloroplast of *A. manshuriensis* (GenBank accession number: NC_046766.1). Gene annotation was corrected with GeSeq (Tillich et al. 2017) and PGA (Qu et al. 2019). A circular chloroplast map was generated using OGDRAW (https://chlorobox.mpimp-golm.mpg.de/OGDraw.html) (Greiner et al. 2019).

Here, we first report and characterize the complete chloroplast of *Aristolochia delavayi* (GenBank accession number: MW413320, this study). The complete chloroplast sequence of *Aristolochia delavayi* possesses a total length of 160,344 bp with the typical quadripartite structure, containing two inverted repeats (IRs) of 25,454 bp separated by a large single-copy (LSC) region and a small single-copy (SSC) region of 89,502 and 197,95 bp, respectively. The chloroplast contains 130 genes, including 85 protein-coding genes, eight ribosomal RNA genes, and 37 tRNA genes (seven of which are duplicated in the IR). The overall G + C% content of the chloroplast was 38.3%. Gene content and gene order are similar to those of *Aristolochia* (Li et al. 2019).

Twelve chloroplast genomes of *Aristolochia* were downloaded from GenBank. *Asarum heterotropoides* (*Asarum*, Aristolochiaceae) was downloaded and included as outgroups (Li et al. 2019). In total, complete chloroplast genome sequences of 13 species were used for the phylogenetic analysis. Sequences were aligned using MAFFT v.7 (Katoh and Standley 2013) and adjusted manually in BioEdit (Hall 1999). ModelFinder (Kalyaanamoorthy et al. 2017) was used to infer the appropriate nucleotide substitution model. GTR + F+I + G4 model was used in the BI and ML analysis based on Bayesian information criterion (BIC). Maximum-likelihood bootstrapping was conducted with 1000 rapid bootstrap (BS) analyses followed by a search for the best-scoring tree in a single run (Stamatakis et al. 2008). Bayesian inference (BI) was conducted for the combined dataset using MrBayes 3.1.2 (Huelsenbeck and Ronquist 2001) with two runs of four Markov chain Monte Carlo chains, each beginning with a random tree and sampling one tree every 1000 generations of 10,000,000 generations. Both ML and BI analyses were conducted on Cipres (Miller et al. 2010). The phylogenetic analysis indicated that *A. delavayi* and *A. tubiflora* are closely related and all clades in *Aristolochia* have maximum support (maximum-likelihood bootstrap support (MLBS)=100 and Bayesian inference posterior probability (BIPP)=1) (Figure 1). The complete chloroplast of *A. delavayi* reported here will provide a useful data for the conservation of the EN species.

**Figure 1.**
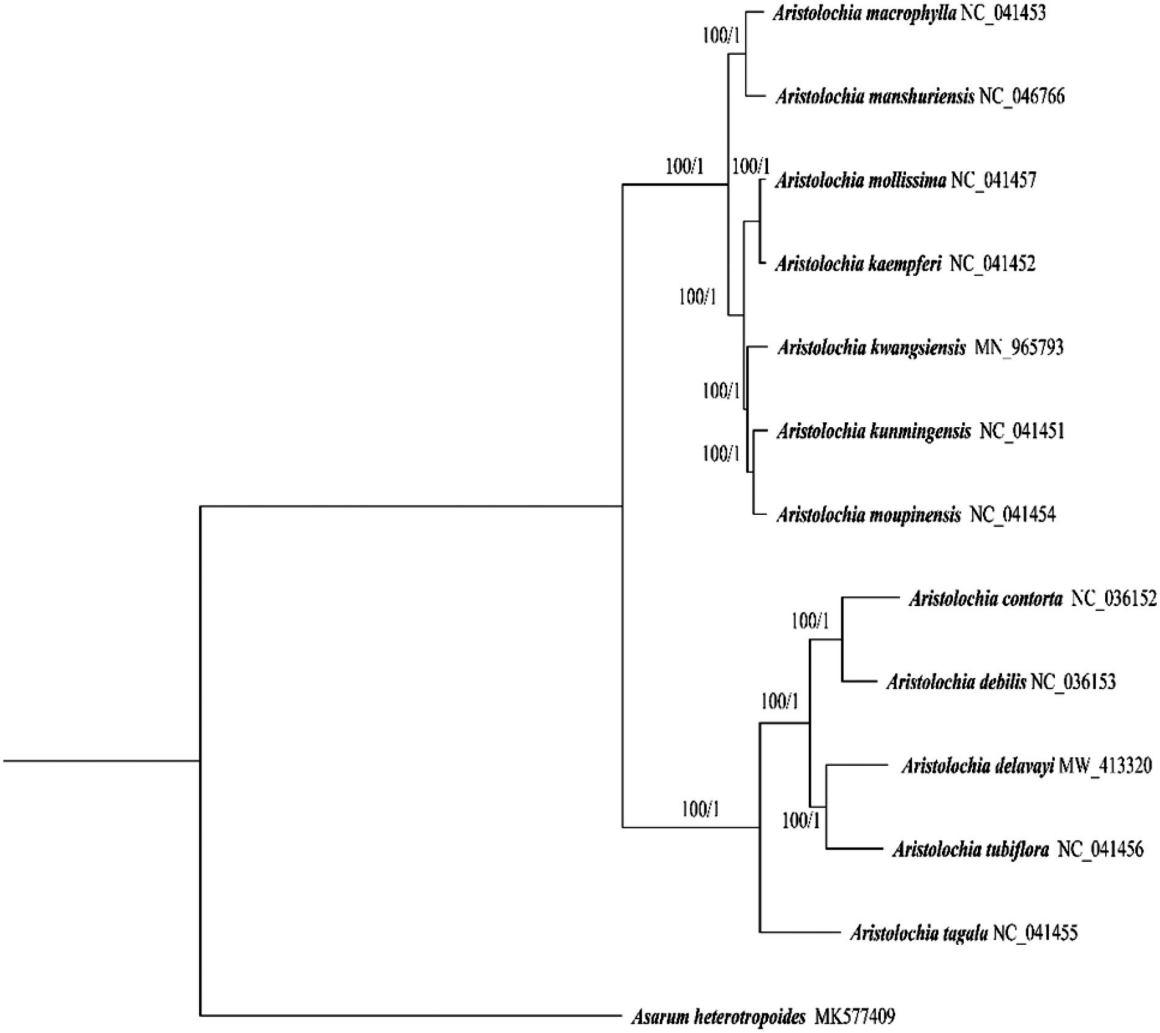
Maximum-likelihood phylogeny of *A. delavayi* and its closely related taxa based on the complete chloroplast genomes. Maximum-likelihood bootstrap support (MLBS) and Bayesian inference posterior probability (BIPP) are given above the branches.

## Data Availability

The data that support the findings of this study are openly available in GenBank of NCBI at https://www.ncbi.nlm.nih.gov, reference number MW413320.
